# Neurodevelopmental delay in children exposed to maternal SARS-CoV-2 in-utero

**DOI:** 10.1038/s41598-024-61918-2

**Published:** 2024-05-24

**Authors:** Viviana Fajardo-Martinez, Fatima Ferreira, Trevon Fuller, Mary Catherine Cambou, Tara Kerin, Sophia Paiola, Thalia Mok, Rashmi Rao, Jyodi Mohole, Ramya Paravastu, Dajie Zhang, Peter Marschik, Sai Iyer, Kalpashri Kesavan, Maria da Conceição Borges Lopes, José Augusto A. Britto, Maria Elisabeth Moreira, Patricia Brasil, Karin Nielsen-Saines

**Affiliations:** 1 David Geffen, UCLA School of Medicine, Los Angeles, CA USA; 2 Universidade do Rio de Janeiro, Rio de Janeiro, RJ Brazil; 3 UCLA Institute for the Environment and Sustainability, Los Angeles, CA USA; 4 Department of Child and Adolescent Psychiatry, Center for Psychosocial Medicine, Heidelberg University, Heidelberg, Germany; 5 Child and Adolescent Psychiatry and Psychotherapy, University Medical Center Göttingen and Leibniz ScienceCampus Primate Cognition, Göttingen, Germany; 6 Interdisciplinary Developmental Neuroscience (IDN), Division of Phoniatrics, Medical University of Graz, Graz, Austria; 7 Fundação Oswaldo Cruz (Fiocruz), Rio de Janeiro, RJ Brazil

**Keywords:** Viral infection, Paediatric research

## Abstract

It is unclear if SARS CoV-2 infection during pregnancy is associated with adverse neurodevelopmental repercussions to infants. We assessed pediatric neurodevelopmental outcomes in children born to mothers with laboratory-confirmed SARS CoV-2 infection during pregnancy. Neurodevelopmental outcomes of in-utero exposed children were compared to that of pre-pandemic control children in Los Angeles (LA), CA, USA and Rio de Janeiro, Brazil. Bayley Scales of Infant and Toddler Development, 3rd edition (Bayley-III), the gold standard tool for evaluating neurodevelopment until 36 months of age and Ages and Stages Questionnaires (ASQ-3), a frequently used screening instrument for evaluating neurodevelopment in this same age group were the assessment tools used. Developmental delay (DD) was defined as having a score < − 2 SD below the norm (< 70) in at least one of three Bayley-III domains, (cognitive, motor or language) or a score below the cut-off (dark zone) in at least one of five ASQ-3 domains (communication, gross motor, fine motor, problem solving, personal-social). Exposed children were born between April 2020 and December 2022 while control children were born between January 2016 to December 2019. Neurodevelopmental testing was performed in 300 children total: 172 COVID-19 exposed children between 5–30 months of age and 128 control children between 6–38 months of age. Bayley-III results demonstrated that 12 of 128 exposed children (9.4%) had DD versus 2 of 128 controls (1.6%), *p* = 0.0007. Eight of 44 additional exposed children had DD on ASQ-3 testing. Fully, 20 of 172 exposed children (11.6%) and 2 of 128 control children (1.6%), *p* = 0.0006 had DD. In Rio, 12% of exposed children versus 2.6% of controls, *p* = 0.02 had DD. In LA, 5.7% of exposed children versus 0 controls, *p* = 0.12 had DD. Severe/critical maternal COVID-19 predicted below average neurodevelopment in the exposed cohort (OR 2.6, 95% CI 1.1–6.4). Children exposed to antenatal COVID-19 have a tenfold higher frequency of DD as compared to controls and should be offered neurodevelopmental follow-up.

## Introduction

SARS-CoV-2 causes adverse pregnancy outcomes worldwide, including maternal mortality and morbidity due to obstetrical complications and preterm delivery^1–4^. Cumulative research studies have correlated *in-utero* exposure to respiratory pathogens in pregnancy and higher risk of future pervasive neurodevelopmental or neuropsychiatric conditions in the offspring; potentially linking maternal immune activation (MIA) as the biological mechanism for these outcomes^5–13^. The role of SARS-CoV-2 infection in pregnancy and long term neurodevelopmental outcomes in the offspring is not well understood. Preterm birth and low birth weight are more prevalent in infants born to pregnant persons with symptomatic COVID-19^14–16^. Prematurity in itself is a risk factor for developmental delay (DD)^17^.

During the COVID-19 pandemic, we initiated a longitudinal observational cohort study of maternal-infant outcomes in pregnancy, the COVID Outcomes in Mother-Infant Pair (COMP) Study, which recruited mother-infant dyads in Los Angeles (LA), CA and Rio de Janeiro (Rio), Brazil, two regions disproportionately affected by the pandemic^18–21^. We utilized the infrastructure and approach implemented by our group during the ZIKV epidemic of 2015–2016^22–26^ to evaluate potential repercussions of COVID-19 in pregnancy, assuming a novel pathogen carries risk to pregnant patients and their offspring. Although some vertically transmitted viruses are knowingly neurotropic, SARS-CoV-2 may potentially be deleterious to progeny through the indirect mechanism of MIA. We hypothesize that MIA, by creating a hostile in utero inflammatory environment during the course of COVID-19 may potentially adversely affect infant neurodevelopment.

In the present analysis, we evaluated neurodevelopmental performance in young children in LA and Rio, using Bayley-III Scales of Infant and Toddler Development, 3rd edition^27^, for assessment of cognitive, motor and language domains. Bayley-III results were contrasted to those obtained in children of comparable age in the immediate pre-pandemic setting. In LA, Ages and Stages Questionnaires (ASQ-3) were performed in a subset of children^28^.

## Results

### Participant characteristics

Between April 2020 and December 2022, 172 children exposed to maternal COVID-19 in pregnancy underwent neurodevelopmental testing. Of 172 children, 128 completed Bayley-III assessments, 44 completed ASQ-3 assessments, and 36 completed both assessments. The cohort was comprised by 97 children aged 11–30 months (1 set of triplets and 2 sets of twins) born to 93 mothers in LA and 75 children (aged 5–28 months) born to 75 mothers in Rio. In addition, 128 pre-pandemic control children of comparable age and demographics underwent Bayley-III testing between January 2017 to December 2019 in Rio, and January 2016 to December 2019 in LA, with a total of 300 evaluable participants. Control children in Rio were recruited during our prior Zika studies and were followed from birth. These control children were selected from healthy pregnancies, had no known evidence of congenital birth defects and had no serological evidence of any congenital infections including Zika virus. All control children recruited in our Zika studies served as controls in the present study. Control children from LA were selected from our Developmental Pediatric clinic and also underwent Bayley-III testing between 2016 and 2019. Control children in LA were selected from healthy pregnancies but had a history of admission to the Neonatal Intensive Care Unit (NICU) following birth, thus being followed in our developmental clinic. Control children in LA were excluded if they had any congenital malformations, or a history of congenital infections. Equal number of preterm controls were matched to our cases for both cohorts in this analysis, and cases and controls were matched by age and gender.

Table 1 compares clinical and demographic parameters between cases and controls. As seen in Table 1, cases and control children were comparable, although a higher rate of premature infants was seen among the control group (20% vs. 32%, *p*-value of 0.03); likely because in LA, our controls were selected from the developmental pediatric clinic.

**Table 1:** Comparison of maternal demographic variables and obstetric and neonatal outcomes between SARS-CoV-2 exposed cases and controls.

Variable		SARS-CoV-2 Exposed	Controls	Total	*p*
Maternal age (years)	< 20	7/165 (4%)	10/125 (8%)	17/273 (6%)	0.21
	20–34	105/165 (64%)	73/125 (58%)	178/273 (65%)	0.4
	> = 35	53/165 (32%)	42/125 (34%)	95/273 (35%)	0.8
SGA		10/171 (6%)	11/128 (9%)	21/299 (7%)	0.37
Prematurity		35/171 (20%)	41/128 (32%)	76/299 (25%)	**0.03**
LBW		31/171 (18%)	25/128 (20%)	56/299 (19%)	0.77
Female sex		84/171 (49%)	62/128 (48%)	146/299 (49%)	0.99
Maternal education	Primary	9/99 (9%)	6/117 (5%)	15/216 (7%)	0.29
	Secondary	63/99 (64%)	57/117 (49%)	120/216 (56%)	**0.03**
	University	27/99 (27%)	54/117 (46%)	81/216 (38%)	**0.005**

Significant values are in bold.

As seen in Table 2, there were significant differences between participants enrolled in Rio and LA. Mothers in LA tended to be older and have diverse racial/ethnic backgrounds. In Rio, 72% of mothers were Black or mixed racial/ethnic backgrounds and all participants had government sponsored health care (*p* < 0.001). As seen in Table 2, LA mothers had a higher frequency of co-morbidities. No participants in Rio were vaccinated before COVID-19, while 30.4% of LA women had received COVID-19 vaccination before infection, *p* < 0.001. In parallel, 8.8% of mothers in LA had severe COVID-19 versus 34.6% of mothers in Rio, *p* < 0.001. Infant outcomes were similar for both groups except for more NICU admissions in LA (21.6% vs. 5.3%, *p* = 0.002) likely reflecting NICU access. In total, 20.5% of infants were preterm, with no differences between sites (Table 2).

**Table 2:** Demographics of pregnant participants infected with SARS-CoV-2 and obstetric/neonatal outcomes.

	LA N = 93	RJ N = 75	*p*
Maternal demographics			
Maternal age, median (range)	34 (18–42)	28 (16–41)	< 0.001
Maternal race/ethnicity (N = 91 LA, N = 68 RJ)			< 0.001
Asian or other, N (%)	23 (25.3%)	0	
Black or mixed race, N (%)	2 (2.2%)	54 (72%)	
US Hispanic, N (%)	40 (44%)	n/a	
White, N (%)	26 (28.6%)	14 (18.7%)	
Maternal education (N = 32 LA, N = 73 RJ)			< 0.001
Primary school	2 (6.3%)	7 (9.3%)	
Secondary school	2 (6.3%)	61 (81.4%)	
Higher education	28 (87.5%)	5 (6.7%)	
Public insurance, N (%) (N = 93 LA 75 RJ)	22 (23.7%)	75 (100%)	< 0.001
Healthcare worker, N (%) (N = 93 75 RJ)	11 (11.8%)	3 (4%)	0.09
Gravida, median (IQR) (N = 92 LA, 73 RJ)	2 (1–3)	2 (1–4)	0.15
Maternal medical history pre-pregnancy			
Maternal medical conditions, N (%) (N = 93 LA, N = 74 RJ)	50 (53.8%)	29 (38.7%)	0.064
Maternal Hx mental conditions, N (%) (N = 93 LA, N = 74 RJ)	20 (21.5%)	2 (2.7%)	< 0.001
Obesity (pre-pregnancy BMI > 30), N (%) (N = 90 LA, N = 65 RJ)	23 (25.6%)	8 (10.7%)	0.045
Diabetes mellitus (not gestational), N (%) (N = 91 LA, N = 74 RJ)	2 (2.2%)	4 (5.3%)	0.41
Pulmonary arterial hypertension, N (%) (N = 89 LA, N = 74 RJ)	1 (1.1%)	5 (6.7%)	0.09
Congenital heart disease, N (%) (N = 90 LA, N = 75 RJ)	2 (2.2%)	0	0.5
Asthma, N (%) (N = 91 LA, N = 74 RJ)	9 (9.9%)	4 (5.3%)	0.39
Autoimmune disorders, N (%) (N = 91 LA, N = 75 RJ)	8 (8.8%)	0	0.008
HIV, N (%) (N = 91 LA, N = 75 RJ)	2 (2.2%)	0	0.5
Obstetric and Neonatal outcomes			
Gestational age at diagnosis (N = 93 LA, 74 RJ)			0.78
1st trimester, N (%)	11 (11.8%)	11 (14.6%)	
2nd trimester, N (%)	30 (32.3%)	21 (28%)	
3rd trimester, N (%)	52 (56%)	42 (56%)	
Median gestational age at delivery in weeks (IQR) (N = 96 LA, 74 RJ)	39 (37–40)	38 (37–39)	0.69
Maternal disease severity (N = 91 LA, 74 RJ)			< 0.001
Asymptomatic, Mild, Moderate, N (%)	83 (91.2%)	48 (64%)	
Severe or Critical, N (%)	8 (8.8%)	26 (34.6%)	
Maternal fever during COVID, N (%) (N = 87 LA, 73 RJ)	25 (28.7%)	31 (41.3%)	0.1
Mode of delivery (N = 93 LA, N = 72 RJ)			0.11
Vaginal, N(%)	59 (63.4%)	36 (48%)	
C-section, N(%)	34 (36.6%)	36 (48%)	
Maternal vaccination before delivery, N (%) (N = 92 LA, 74 RJ)	31 (33.7%)	13 (17.3%)	0.03
Maternal vaccination before infection, N (%) (N = 92 LA, 75 RJ)	28 (30.4%)	0	< 0.001
Sex assigned at birth female, N (%) (N = 97 LA, 74 RJ)	51 (52.6%)	33 (44%)	0.35
Neonatal complications N (%) (N = 97 LA, 74 RJ)			
Preterm delivery	21 (21.6%)	14 (19%)	0.71
Small-for-gestational-age,	4 (4.1%)	6 (8%)	0.33
Low birth weight (< 2500 g)	21 (21.6%)	10 (13.3%)	0.22
Respiratory distress syndrome	14 (14.4%)	5 (5.3%)	0.14
Neonatal intensive care unit admission	21 (21.6%)	4 (5.3%)	0.002

Maternal medical conditions included hypertensive disorders of pregnancy, Diabetes mellitus (including gestational diabetes), and pre-pregnancy obesity.

#### Prevalence of neurodevelopmental outcomes

**Figure 1:**
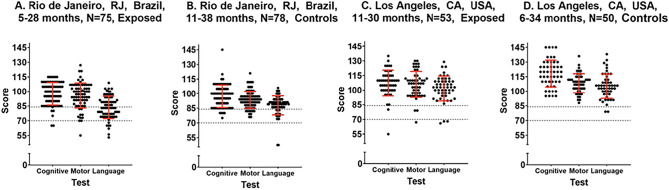
Bayley-III assessments for children exposed to maternal COVID-19 in Rio de Janeiro, RJ, Brazil (n = 75) and Los Angeles (n = 53), CA, USA [n = 128] compared to pre-pandemic control children from Rio de Janeiro (n = 78) and Los Angeles (n = 50), [n = 128]. Total = 256. Distribution of Bayley-III scores for cognitive, motor and language domains among 128 case and 128 control children in Rio de Janeiro, Brazil and Los Angeles, CA. Scores < 85 to 70 are between − 1 and − 2 SD and reflect risk of developmental delay. Scores < 70 are less than − 2 SD and reflect developmental delay.

**Figure 2:**
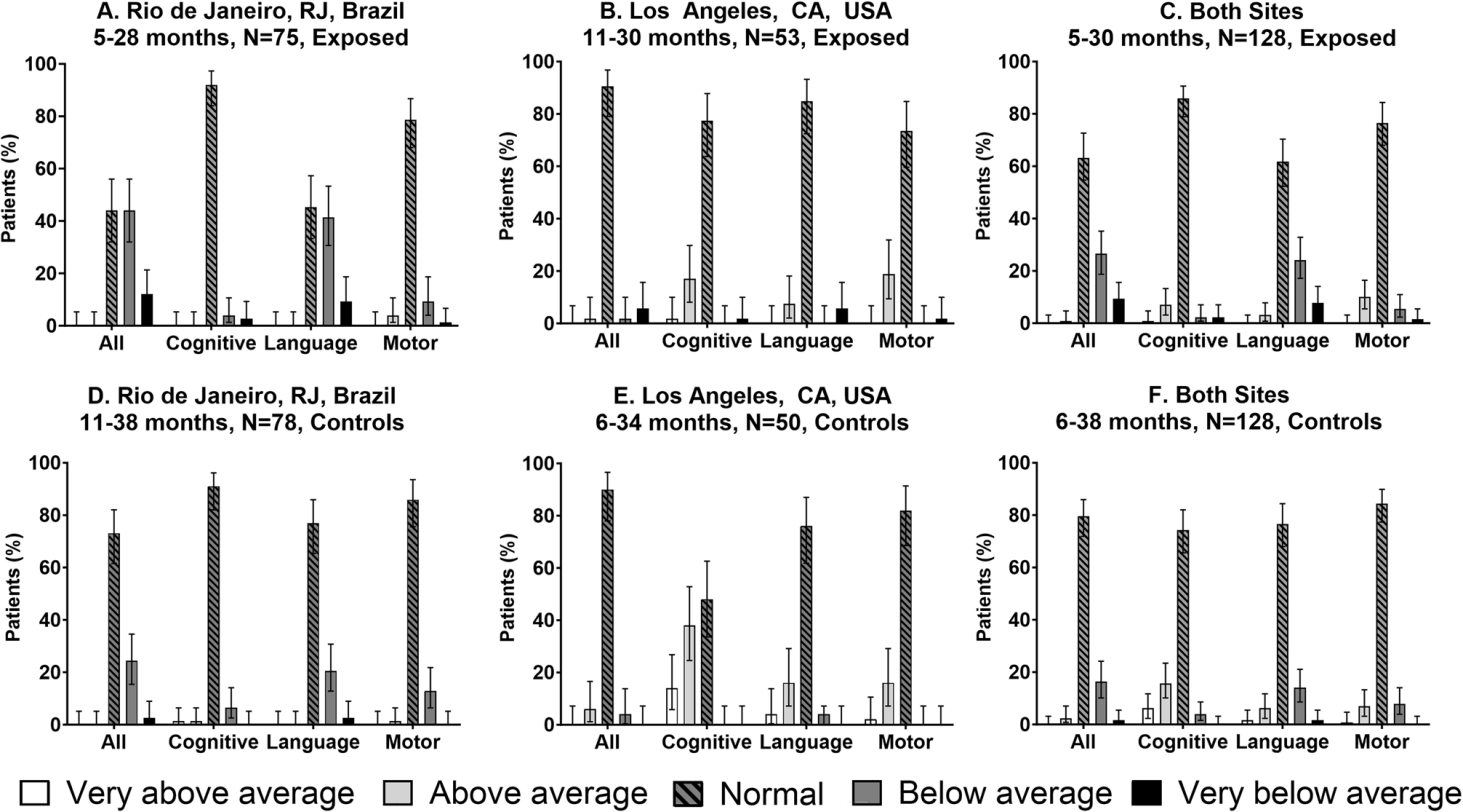
Distribution of Bayley-III results in children exposed to maternal COVID-19 in pregnancy and pre-pandemic healthy control children in Rio de Janeiro and Los Angeles.

In the LA cohort, Bayley-III median composite scores for cognitive, language and motor domains in cases and controls respectively were 110 and 120, *p* < 0.001, 103 and 106, *p* = 0.31 and 107 and 110, *p* = 0.15 (Fig. 1). In the Rio cohort, Bayley-III median composite scores for the cognitive domain in cases versus controls were 100 and 98, *p* = 0.8, in the language domain were 83 and 89, *p* = 0.01 and in the motor domain were 97 and 94, *p* = 0.21. The COVID-19 cohort tended to have more children with developmental delay (< − 2 SD) as compared to pre-pandemic controls (Fig. 2). In addition, in Rio, 9 of 75 children (12%) were delayed in the COVID-19 cohort, as opposed to 2 of 78 children (2.6%) in the control group, *p* = 0.02. In LA, 3 of 53 children (5.7%) were delayed as compared to none in the control group, *p* = 0.12. The lower scores were driven primarily by the language domain. In Rio, a higher number of children (n = 33, 44%) were found to be at rDD in the COVID-19 cohort versus the control group (n = 19, 24.3%), *p* = 0.01. Only one child was at rDD in LA versus 2 controls (Table 3). Overall, combining Bayley-III results for Rio and LA, 12 of 128 children (9.4%) exposed to maternal COVID-19 had scores below 70, indicative of severe DD, as compared to 2 of 128 control children (1.6%), *p* = 0.007 (Table 3).

**Table 3:** Distribution of Bayley-III results in children exposed to maternal COVID-19 in pregnancy and pre-pandemic healthy control children in Rio de Janeiro and Los Angeles.

		At risk for developmental delay (< − 1 and ≥ − 2 SD), n (%)		*p*	Developmentally delayed (< − 2 SD), n (%)		*p*
		Exposed*	Controls**		Exposed*	Controls**	
Rio de Janeiro	All	33 (44.0%)	19 (24.3%)	**0.01**	9 (12.0%)	2 (2.6%)	**0.02**
	Cognition	3 (4%)	5 (6.4%)	0.54	2 (2.7%)	0	0.24
	Language	31 (41.3%)	16 (20.5%)	**0.006**	7 (9.3%)	2 (2.6%)	0.08
	Motor	7 (9.3%)	10 (12.8%)	0.53	1 (1.3%)	0	0.24
Los Angeles	All	1 (1.9%)	2 (4.0%)	0.60	3 (5.7%)	0	0.12
	Cognition	0	0	0.99	1 (1.9%)	0	0.50
	Language	0	2 (4.0%)	0.17	3 (5.7%)	0	0.12
	Motor	1 (1.9%)	0	0.50	1 (1.9%)	0	0.50
Both sites	All	34 (26.6%)	21 (16.4%)	0.05	12 (9.4%)	2 (1.6%)	**0.007**
	Cognition	3 (2.3%)	5 (3.9%)	0.05	3 (2.3%)	0	0.12
	Language	32 (24.2%)	18 (14.1%)	**0.04**	10 (7.8%)	2 (1.6%)	**0.021**
	Motor	8 (6.3%)	10 (7.8%)	0.68	2 (1.6%)	0	0.50

*53 children were exposed to maternal COVID in Los Angeles, 75 in Rio de Janeiro, total: 128.

**50 children were pre-pandemic controls in Los Angeles, 78 in Rio de Janeiro, total: 12.

Significant values are in bold.

**Figure 3:**
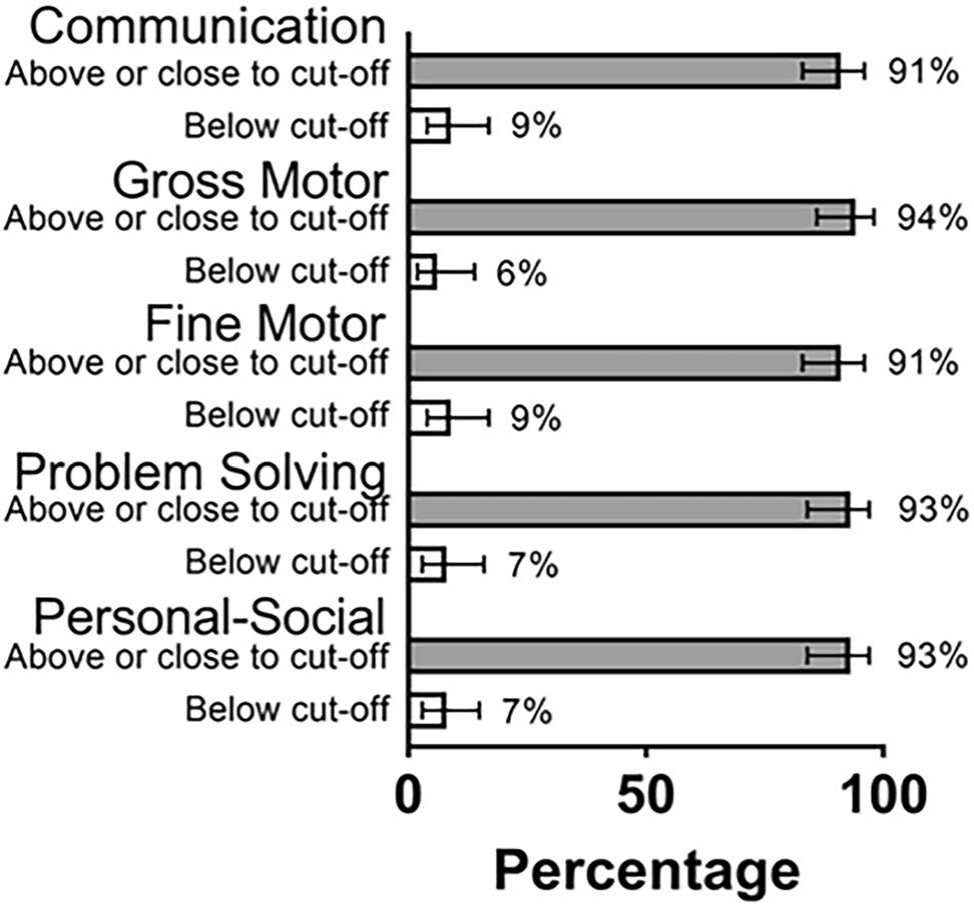
ASQ-3 assessments for 80 children exposed to maternal COVID-19 in utero (age 4–28 months), Los Angeles, CA. Distribution of ASQ-3 results for children born to mothers with COVID-19 in Los Angeles, CA for communication, gross motor, fine motor, problem solving and personal-social domains.

Among 80 children with ASQ-3, 6% to 9% scored below the cut-off for the age-appropriate assessment (Fig. 3). The higher frequency of abnormal scores were in language and fine motor domains. Forty-four children had ASQ-3 performed as their only neurodevelopmental assessment, 8 children (18.2%) scored below the cut-off in at least one domain. Thirty-six children had both ASQ-3 and Bayley-III at the same visit, allowing comparison of results. The AUC for the ASQ-3 as a predictor of rDD or DD according to Bayley-III ranged from 0.84 to 0.9 and was statistically significant for all domains (Cognitive: 0.844 95% CI 0.659–1, *p* = 0.027, Language: 0.844 95% CI 0.689–0.99, *p* = 0.05, Motor: 0.903, 95% CI 0.799–1, *p* = 0.023), indicating performance was similar in both tests with ASQ-3 adequately predicting developmental outcomes when compared to the gold standard. In total, 20 (12 via Bayley-III and 8 via ASQ-3) of 172 children (11.6%) had DD.

#### Association between maternal characteristics and outcomes

**Figure 4:**
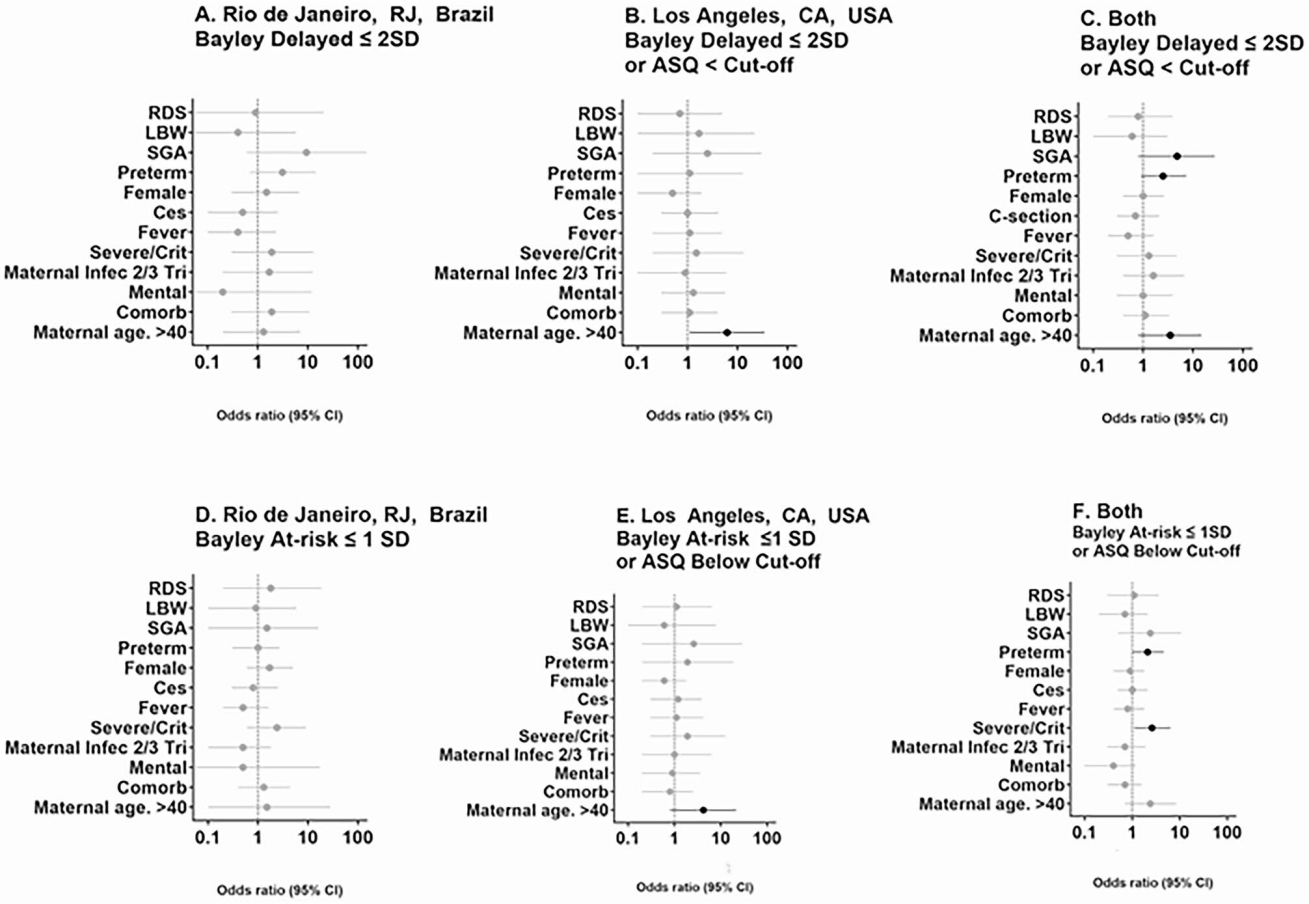
Predictors of below average neurodevelopment (developmental delay and risk of delay) in COVID-19 exposed infants. Graphic representation of logistic regression analysis of potential variables associated with developmental delay (DD) on Bayley-III testing (< − 2 SD or score < 70 in any of the 3 functional domains) or an ASQ-3 assessment below the cut-off (if Bayley-III was not done) for children exposed to maternal COVID-19 in Rio de Janeiro (**A**), Los Angeles (**B**) and both sites combined (**C**). (**D**–**F**) include children at risk for DD and children with DD on Bayley 3 testing (< − 1 SD/scores < 85) or an ASQ-3 below the cut-off. Children in Rio de Janeiro are depicted in (**D**), children from LA in (**E**) and both sites are shown in (**F**). LBW: low birth weight; SGA: small for gestational age; Vacc: maternal vaccination in pregnancy; Ces: C-section delivery; Fever: maternal fever due to COVID-19 in pregnancy; Severe/Crit: severe or critical COVID-19 in pregnancy; Maternal Infec 2/3 Tri: maternal COVID-19 in the 2nd or 3rd trimester of pregnancy; Mental: mental co-morbidities; Comorb: comorbidities as defined in Table 2. Individual details for the panels and numerical variables are provided in the Supplemental Tables.

Potential predictors of DD were evaluated in children exposed to antenatal COVID-19 (Fig. 4 and Supplemental Tables). The only predictor of DD in LA was maternal age greater than 40 years of age (OR 6.2, 95% CI 1.1–34.8). No predictors were identified in Rio. Maternal age (OR 3.5, 95% CI 0.8–14.8), small for gestational age (OR 4.8, 95% CI 0.8–27.2) and preterm delivery (OR 2.5, 95% CI 0.9–7.3) trended towards significance for DD with both sites combined (Fig. 4). When the analysis was performed for predictors of below average performance (rDD and DD in Bayley-III and below cut-off in ASQ-3), for both sites combined, significance was noted for severe/critical maternal COVID-19 (OR 2.6, 95% CI 1.1–6.4). Preterm delivery trended towards significance but did not achieve it as predictor of below average performance (OR 2.1, 95% CI 1–4.6). Interestingly, among the 12 children who had DD on Bayley-III testing, only 2 were preterm (16.7%); 1 at each site. Three of 8 children (37.5%) who scored below the ASQ-3 cut-off in LA were preterm.

## Discussion

Earlier findings from our longitudinal cohort demonstrated a pattern of deviant early neuromotor functions and neurodevelopmental capacities in our study population. Similarly, early screening for the integrity of neuromotor development in our infants through the General Movements Assessment (GMA) demonstrated an abnormal endogenous movement character at 3 to 5 months of age, a sign of sub-optimal nervous system functioning in 14% of our COVID-19 cohort as compared to 0% in pre-pandemic controls^20^. Additionally, delay in attainment of developmental milestones was identified in 12% of children between 6 and 8 months of age^20^. In the present analysis, 9.4% of children exposed to maternal COVID-19 had composite scores below 70 in at least one domain on Bayley-III, indicative of DD, as compared to 1.6% of pre-pandemic control children from the same environment, a statistically significant finding. Taking into account both assessment tools (Bayley-III and ASQ-3), 12% of 172 exposed children in both cohorts (LA and Rio) had DD. Our COMP study data continues to demonstrate higher frequencies of DD in children exposed to maternal COVID-19 at different ages evaluated with distinct tools in the first months of life as previously published^20^ and in the first three years of life as shown in this study. In healthy control populations, this degree of DD is fairly uncommon^27^.

Although transplacental transmission of SARS-CoV-2 is rare^29^, the virus may trigger MIA and sometimes, a cytokine storm^30^. COVID-19 immune dysregulation in pregnancy, a physiologic condition normally marked by trimester-specific T-cell regulation, augments systemic inflammation risk^31^. MIA has been implicated in the pathogenesis of infant central nervous system disorders, with data suggesting higher risk of future pervasive neurodevelopmental or neuropsychiatric conditions such as autism spectrum disorder, schizophrenia, and cerebral palsy among many neurodevelopmental problems^5,6,32–36^. Research indicates MIA affects fetal brain development with changes in brain structure and function, inducing neuronal dysfunction and divergent behavioral phenotypes^6,37,38^. Proteomic results from our COMP cohort of pregnant mothers with severe disease and their infants showed evidence of significant maternal immune activation and abnormal Wnt/b-catenin signaling among newborns^18^, a canonical pathway associated with pervasive neurodevelopmental disorders^39^. MIA could potentinally be a mechanism leading to abnormal neurodevelopmental outcomes; however, further research is needed.

Our findings are supported by that of others, who demonstrated, through different approaches, a higher neurodevelopmental risk among children born to mothers with COVID-19. In a large series evaluating ICD-10 diagnostic codes over the first year of life in 7772 live births, SARS-CoV-2 in pregnancy was associated with a greater frequency of neurodevelopmental diagnoses^40^. A second study using the same approach for 18,355 live births demonstrated a higher risk of neurodevelopmental diagnoses in male infants at 12 months of age, but not at 18 months^41^. We did not observe sex to be a risk factor for DD in our cohort. A study of 255 infants enrolled in the COVID-19 Mother Baby Outcomes Initiative using ASQ-3 as the neurodevelopmental tool at 6 months of age, demonstrated that infants born during the pandemic had significantly lower scores on gross motor, fine motor and personal-social subdomains than historical controls^42^. A study using a telehealth tool similar to ASQ-3 evaluated 407 SARS-CoV-2 exposed and unexposed infants in New York without finding associations between prenatal SARS-CoV-2 exposure and infant neurodevelopment when mothers had asymptomatic or mild disease^43^.

To our knowledge, our study is the first to evaluate children with antenatal SARS-CoV-2 exposure using Bayley-III as the primary neurodevelopmental tool, which is considered the “gold standard” for assessing neurodevelopment in this age group. Questionnaire-based evaluations such as the ASQ-3 are validated in clinical practice as screening tools for assessing infants at risk for adverse developmental outcomes. When children screen positive, they are referred to in-person detailed assessments such as Bayley-III. Being a screening tool, ASQ-3, which we used in study participants who could not come to clinic, has higher sensitivity but lower specificity, whereas Bayley-III is more specific for DD^44^. We found comparable results for both assessments among children evaluated by both methods on the same day.

Our results demonstrate that the most affected domain among exposed infants was language development. There is considerable debate in the literature whether pandemic lockdowns, lack of interaction between parents and children, parental depression and the use of masks may be responsible for children having below average neurodevelopmental performance across several studies. A study that followed children from 1 to 3 years and 3 to 5 years of age, observed that pandemic-exposed children were more prone to delayed childhood development by age 5 years^45^. It is unquestionable that infant stimulation and socio-economic factors play a very important role in infant neurodevelopment. We observed that below average Bayley-III scores (rDD) above the DD threshold (< − 2SD) were high in Rio, where social and economic inequities prevailed among participants, but were not prevalent in LA. It is likely that below average performance (rDD category) can be explained by pandemic circumstances; however, a score less than 70 in any domain demonstrates severe DD, requiring further mechanistic investigation. Our own proteomic analyses and that of others suggested possible pathways ranging from MIA by SARS-CoV-2^18^, maternal fever, placental thrombosis with fetal ischemia^18,20^, and other adverse early infant outcomes^46^ as explanations for delayed neurodevelopment. COVID-19 has been shown to induce preterm birth^1^, including our study which had a prematurity rate of 20% as compared to 10.4% in the general population^47^. Preterm infants are known to be at higher risk for poor neurodevelopmental outcomes^17^. However, only 2 of 12 children with DD on Bayley-III were preterm, suggesting other potential mechanisms may be at play. A higher prevalance of prematurity among control children renders prematurity an unlikely cause of higher DD rates in our exposed infants. In addition the assessments utilized in this analysis (Bayley-III and ASQ-3) adjust for gestational age at birth. One other important observation is the higher prevalance of secondary education in cases as opposed to controls. Although mothers of controls were more likely to have completed higher education (46%) as compared to cases (27%), the severity of DD noted (< − 2SD) makes it unlikely that maternal education alone could explan this extent of DD.

We could not identify predictors of DD associated with COVID-19 exposure for both sites, beyond COVID-19 exposure itself. In LA, older maternal age was a predictor. Severe/critical maternal COVID-19 was associated with below average neurodevelopmental performance at both sites (rDD and DD). Advantages of our study are that a universally accepted “gold standard” neurodevelopmental tool was used, and SARS-CoV-2 maternal exposure was laboratory confirmed. A study limitation is that our controls were recruited in the pre-pandemic period, so we cannot rule out the pandemic scenario as a trigger of DD, although we find this unlikely given the severity of this finding. DD with Bayley-III scores less than -2 SD was the degree of DD noted in microcephalic Zika virus exposed children in our prior studies^25^. We could not recruit concurrent control children because of high rates of COVID-19 in pregnancy, rendering it nearly impossible to exclude exposure among controls.

In summary, children exposed to antenatal COVID-19 have higher frequencies of DD as compared to non-exposed controls. Risk factors for DD beyond maternal COVID-19 were not identified across sites; severe maternal disease was predictive of below average neurodevelopment. Long-term neurodevelopmental follow-up should be considered in children exposed to antenatal COVID-19. Understanding of mechanistic pathways triggering DD should be investigated.

## Methods

The study was approved by the institutional review boards of the University of California, Los Angeles (UCLA), U.S. and Fundação Oswaldo Cruz (Fiocruz) in Rio, Brazil, and was carried out in accordance with the Declaration of Helsinki. Informed consent was obtained from parents.

### Study design

This was an observational cohort with two groups of children. The study followed the Strengthening the Reporting of Observational Studies in Epidemiology (STROBE) guidelines. The first group of children were participants in the COVID-19 Outcomes in Mother-Infant Pairs (COMP Study), a longitudinal observational cohort study, of maternal-infant outcomes in pregnancy^19,20,48,49^. Standard study procedures have been previously described^19,20^. Inclusion criteria for study participation required PCR-confirmed maternal SARS CoV-2 infection in pregnancy, prenatal care and delivery at one of the participating sites in Rio or LA, and willingness to consent for study participation and infant follow-up at one of the participating sites. Exclusion criteria included no laboratory documentation of SARS-CoV-2 infection during pregnancy, presence of congenital birth defects, and/ or parental unwillingness to provide informed consent, or inability to bring their children to study visits for the length of the study (birth to 3 years). Participants were recruited in the outpatient obstetric clinic and labor and delivery units at UCLA and from a maternity hospital in Caxias, Rio de Janeiro, Brazil. All mothers were identified with SARS-CoV-2 infection via reverse transcriptase polymerase chain reaction of naso-pharyngeal specimens. Infants were similarly screened for SARS-CoV-2 within 48 h of life if mother was positive at delivery. Infants were followed from birth and every 6 months thereafter with in person visits to our clinic. Exposed infants were recruited in LA and Rio from 2020 to 2022. The pre-pandemic control group of children originated from two different study groups. Control children in Rio were healthy children unexposed to Zika virus from from our prior Zika studies, the Zika study cohort^22,50–52^. All Rio control children were recruited and followed at the same institution in Rio de Janeiro where the COVID-19 exposed children were recruited. These children were born between 2017 and 2019 and followed from birth. They were the product of normal pregnancies, and were of similar age, sex and age at performance of developmental assessment as COVID-19 exposed children from the same institution. Control children in Rio had no evidence of congenital birth defects and no clinical or laboratory evidence of congenital infections including Zika virus. The second group of control children, from Los Angeles, was selected from our Developmental Pediatric clinic at UCLA, also the same institution from where LA exposed children were recruited. Control children in LA were selected from healthy pregnancies but had a history of admission to the Neonatal Intensive Care Unit (NICU), thus being followed in our developmental clinic from the time of birth between 2016 and 2019. Fifty control children out of 400 patients followed in the clinic were selected based on compliance with in person follow-up visits, availability of Bayley-III neurodevelopmental assessments and performance of these assessments at the same institution. Control children were excluded if they had any congenital birth defects or any medical history of congenital infections. Control groups were matched to cases by gestational age, gender and age at the time of neurodevelopmental testing.

### Setting

The study was conducted at pediatric hospitals affiliated with UCLA (LA) and FIOCRUZ (Rio), both academic institutions. Children were allocated into two groups- exposed versus control groups with neurodevelopmental testing performed for both groups at both sites. For the exposed group, mother-infant pairs in the COMP study were enrolled from April 4, 2020, to December 18, 2022. For the control group, children from Rio were enrolled from January 2017 to December 2019; and children in LA were recruited from January 2016 to December 2019. Bayley-III testing was performed until September 2023. Children without antenatal SARS-CoV-2 exposure were selected from prior Zika studies in Rio and from our Developmental Pediatric clinic in LA.

### Participants

Three hundred children were enrolled. Infants exposed to maternal SARS-CoV-2 (n = 172) were born between April 4, 2020, to December 18, 2022, in Rio (n = 75) or LA (n = 97). Control children (n = 128) were born between January 2017 to December 2019 in Rio and between January 2016 to December 2019 in LA. Both groups were followed prospectively since the time of birth. Control children were enrolled if they matched exposed children in age, sex and age of performance of the neurodevelopmental assessment*.* Equal number of preterm controls were matched to preterm cases for both cohorts. Although, contemporary controls would have been ideal, most mothers in the COMP study became pregnant at the height of the pandemic, within the first 2.5 years mainly; it was impossible during that time to guarantee pregnant women in the control group had not had COVID-19 during gestation as antibody screening proved unreliable. Therefore, the use of controls unexposed to the virus in question from the pre-pandemic period enables a comparator group that definitely does not have the exposure in question. The control groups for Rio (n = 78) and LA (n = 50) were chosen because they were both recruited and followed at the same institutions at both sites and were representative of the exposed study population. Controls were followed by the same study investigators who subsequently followed the cases, using the same developmental protocols, with evaluations administered by the same study personnel. Their developmental evaluations were performed not too distant in time from that of the exposed children. In addition, they were of similar gestational age, sex and age at the time of performance of the developmental assessments, and none of them had the exposure in question.

### Variables

Children were enrolled in the exposure category if mothers had a positive RT-PCR for SARS-CoV-2. Exposed children were matched to children of comparable age, gender and approximate age at time of Bayley-III performance to unexposed children tested before the pandemic. Seventy-five children in Rio and 53 in LA in the exposure category (n = 128) had Bayley-III assessments. Eighty children in the exposure category in LA had ASQ-3 performed; for 44 children ASQ-3 was the only assessment. Data on maternal COVID-19 severity was collected at enrollment using U.S. National Institutes of Health (NIH) COVID-19 treatment guidelines^53^. Details of data collection were previously reported^19,20,48^. Further details are provided in the supplement.

### Neurodevelopmental assessments

Bayley-III^27^ was chosen as the main neurodevelopmental instrument because it is the gold standard for neurodevelopmental testing in children of this age group and is validated cross-culturally in Brazil^54^. ASQ-3^28^ were performed in 80 children in LA, 36 of whom also had Bayley-III assessments the same day. DD was defined as a composite score < − 2 SD (< 70); risk of DD (rDD) composite score < − 1 SD ≥ − 2 SD (< 85–70) measured by Bayley-III assessments in any of the three domains (cognitive, language and motor) in cases and controls; scores below ASQ-3 cut-offs in at least one of the five domains were considered consistent with DD. When children had more than one Bayley-III assessment, the older age bracket was used in the analysis. When LA children had both Bayley-III and ASQ-3 assessments, Bayley-III was used in the analysis. Seventy-eight children in Rio and fifty children in LA had Bayley-III (n = 128) performed in the pre-pandemic period. Control children came from uncomplicated pregnancies, with congenital infections and genetic diseases ruled out by serologic evaluations, newborn screening and frequent clinical examinations. Details of neurodevelopmental assessments are provided in the supplement.

### Data sources and management

Bayley-III assessments for children with exposure to maternal illness during pregnancy were conducted prospectively by study personnel in Brazil and the U.S. with results recorded in case report forms (CRFs). Children without exposure undergoing Bayley-III testing were evaluated in perinatal clinics at the same institutions before the pandemic. ASQ-3 assessments in LA were performed by study personnel who interviewed parents, with results recorded in CRFs. Study protocol for Bayley-III assessments were performed starting at 12 months and yearly after for the first 3 years of life in the LA cohort. In Rio, Bayley-III assessments were started earlier than 12 months of age and performed every 6 months thereafter for the first 3 years of life.

### Bias

Study personnel performing Bayley-III and ASQ-3 assessments were aware of antenatal maternal SARS-CoV-2 exposure, as were parents responding to ASQ-3 s. All neurodevelopmental tools were standardized, and scores were provided according to testing guidelines.

### Study size

One hundred seventy-two children were enrolled in the exposure group; this was the number of children tested. In LA the age of the participants at the time of evaluation ranged from 11 to 30 months of age and in Rio from 5 to 28 months of age. One hundred twenty-eight children of comparable age and demographics were enrolled in the control group. Children undergoing Bayley-III testing in the exposure group (n = 128) were compared to 128 children in the control group. Eighty children had ASQ-3 assessments, including 44 children with no Bayley-III evaluations, comprising 300 total participants.

### Statistical analysis

Pearson χ2 test or Fisher’s exact test was used to compare Bayley-III scores (categorical data) of exposed infants versus controls. Bayley-III scores were compared between exposed and control children within Rio and within LA. Comparisons of median Bayley-III scores were analyzed by the Mann–Whitney U test for comparing two groups and Kruskal–Wallis for comparing multiple groups. Obstetrical and neonatal outcomes among exposed children in LA and Rio were compared using Fisher’s exact test. Logistic regression was performed to evaluate potential predictors of DD among exposed children in Rio and LA, and children scoring below average; rDD and DD (< − 1 SD or ASQ-3 below the cut-off). Predictor variables included low birth weight, preterm delivery, infant female sex, C-section delivery, maternal fever in pregnancy, severe/critical maternal COVID-19, maternal 2nd or 3rd trimester infection, maternal mental illness or comorbidities, and maternal age > 40 years. COVID-19 severity (asymptomatic = 0, mild/moderate = 1, severe/critical = 2), trimester of infection (first, second, third) and maternal age were analyzed as continuous variables, with odds ratios predicting risk with each increasing unit. Other variables (fetal sex-reference: female; neonatal and maternal comorbidities—reference: none; maternal fever with COVID-19—reference: none; preterm birth—reference: none) were classified as dichotomous. Analysis was done with simple logistic regression for each predictor variable, subsequently all variables were included in a full model for potential confounding effects.

When children had both Bayley-III and ASQ-3 results available, Bayley-III results were used in the analysis. To assess concordance between ASQ-3 and Bayley-III tests, we calculated the area under the curve (AUC) of the receiver operator characteristic curve (ROC)^55^. For this calculation, the outcome was being rDD or DD on Bayley-III. We calculated the statistical significance of the AUC using SPSS. Two-sided *p* < 0.05 was considered statistically significant. Data was analyzed using SAS V.9.4. Further details are provided in the supplement.

### Supplementary Information

Supplementary Information.

## Data Availability

Deidentified data are available upon reasonable request.
