# Supplementary material for: Neurodevelopmental delay in children exposed to maternal SARS-CoV-2 in-utero
Source: Sci Rep. 2024 May 24;14:11851. doi: 10.1038/s41598-024-61918-2 (PMC11126599; doi:10.1038/s41598-024-61918-2)
Supplement: Supplementary file 1 — Supplementary Information. [file 41598_2024_61918_MOESM1_ESM.docx]

Supplemental materials

Neurodevelopmental testing:

All infants enrolled in the present study had detailed neurologic assessments. Bayley-III was performed by trained personnel (V.F.M in Los Angeles and F.F. in Rio de Janeiro) for three domains: cognitive, language (receptive and expressive) and motor (fine and gross)^1^. Bayley-III was chosen as the developmental tool, as it is validated cross-culturally in Brazil.^2^ Reported results are for the latest Bayley-III assessment performed for each child, ranging from 5 to 30 months of age. A Bayley-III score within 1 s.d. above or below the norm of 100 is considered normal for that specific domain. A score of 1–2 s.d. above the norm (116–130) is considered above average and 1–2 s.d. below the norm (<85 to 70) moderately below average. A score >2 s.d. from the norm (>130) is considered well above average and 2 s.d. below the norm well below average (<70). The scoring system is consistent with previously published developmental studies.^1 -7^ Bayley-III assessments are adjusted for gestational age at delivery and thus adjusted for prematurity.

Ages and Stages Questionnaires (ASQ-3s) are age-appropriate questionnaires given to parents to assess 5 neurodevelopmental domains: communication, gross motor, fine motor, problem solving and personal-social domains^8^. Questionnaires assess the ability the child has to perform a task, with different scores rendered for Yes, Sometimes and Not yet. Cut-off scores vary by age of performance of the assessment and are depicted as below cut-off, at the threshold, and age-appropriate. ASQ-3 corrects for gestational age of delivery (thus adjusted for prematurity) and is considered an appropriate screening neurodevelopmental assessment. Children who perform below the cut-off in ASQ-3 assessments are generally referred for further evaluation using Bayley-3 testing. ASQ-3 assessments in the present study were performed in clinic by V.F.M. and K.N.S. in Los Angeles.

Data collection:

Maternal COVID-19 severity categories were collapsed into: asymptomatic, mild/moderate and severe/critical for analyses. Clinical, obstetrical and laboratory results were abstracted from medical records. Data included: country of enrolment, assigned sex, mode of delivery, preterm delivery (<37+0 weeks and <34+0 weeks), birth weight, APGAR scores (1 and 5 min) and neonatal comorbidities. Maternal characteristics included: COVID-19 disease severity, presence of fever during COVID-19, trimester at diagnosis, multiple versus singleton gestation and maternal comorbidities. These were classified as hypertensive disorders of pregnancy, diabetes mellitus (including gestational DM) or pre-pregnancy obesity body mass index >30 kg/ m2. Maternal mental comorbidities included depression, anxiety or substance use disorder.

References:

1. Bayley, N. Bayley Scales of Infant and Toddler Development: Administration Manual (Harcourt Assessment, 2006).
2. Madaschi, V., Mecca, T. P., Macedo, E. C. & Paula, C. S. Bayley-III Scales of

Infant and Toddler Development: transcultural adaptation and psychometric properties. Paidéia (Ribeirão Preto) 26, 189–197 (2016).

1. da Cunha, R. D. E. S., Lamy Filho, F., Rafael, E. V., Lamy, Z. C. & de Queiroz, A. L. G. Breast milk supplementation and preterm infant development after hospital discharge: a randomized clinical trial. J. Pediatr. 92, 136–142 (2016).
2. Souza, C. T. et al. Assessment of global motor performance and gross and fine motor skills of infants attending day care centers. Rev. Bras. Fisioter. 14, 309–315 (2010).
3. Johnson, S., Moore, T. & Marlow, N. Using the Bayley-III to assesneurodevelopmental delay: which cut-of should be used? Pediatr. Res. 75, 670–674 (2014).
4. Jary, S., Kmita, G. & Whitelaw, A. Diferentiating developmental outcome between infants with severe disability in research studies: the role of Bayley Developmental Quotients. J. Pediatr. 159, 211–4.e1 (2011).
5. Nielsen-Saines, K.*, et al.* Delayed childhood neurodevelopment and neurosensory alterations in the second year of life in a prospective cohort of ZIKV-exposed children. *Nat Med* **25**, 1213-1217 (2019).
6. Squires, J., & Bricker, D. Ages and Stages Questionnaire (ASQ): A Parent Completed Child Monitoring System (3rd ed.). *Baltimore, MD: Brooks Publishing Company.* (2009).

Supplemental Tables^1,2^:

Supplemental Table 1. Title: Predictors of below average neurodevelopment (developmental delay and risk of delay) in COVID-19 exposed infants. Subtables A-F report the values of the Odds Ratios displayed in Figure 3 of the main text, and the corresponding *p*-values.

A. Rio de Janeiro, RJ, Brazil, Bayley Delayed < - 2 SD

| **Variable** | **Odds Ratio (95% CI)** | **p-value** |
| --- | --- | --- |
| Low birth weight (LBW) | 0.4 (0-5.7) | 0.4906 |
| Small for gestational age (SGA) | 9.4 (0.6-148.9) | 0.1133 |
| Preterm delivery (<37 weeks) | 3.1 (0.7-14.3) | 0.1382 |
| Female sex | 1.5 (0.3-6.6) | 0.6221 |
| C-section | 0.5 (0.1-2.5) | 0.3879 |
| Maternal fever during pregnancy | 0.4 (0.1-2.3) | 0.2788 |
| Maternal severe/critical COVID-19 | 1.9 (0.3-12.9) | 0.5295 |
| Maternal SARS-CoV-2 infection during the 2nd or 3rd trimester | 1.7 (0.2-12.4) | 0.5959 |
| Maternal mental health conditions | 0.2 (0-11.9) | 0.4126 |
| Other maternal comorbidities | 1.9 (0.3-10.6) | 0.4526 |
| Maternal age > 40 years | 1.3 (0.2-7) | 0.7768 |
| Infant respiratory distress syndrome (RDS) | 0.9 (0-20.9) | 0.9561 |

B. Los Angeles, CA, USA, Bayley Delayed < - 2SD or ASQ-3 < Cut-off

| **Variable** | **Odds Ratio (95% CI)** | **p-value** |
| --- | --- | --- |
| Low birth weight (LBW) | 1.7 (0.1-21.7) | 0.6982 |
| SGA (Small for gestational age) | 2.5 (0.2-30) | 0.4594 |
| Preterm delivery (<37 weeks) | 1.1 (0.1-12.8) | 0.9314 |
| Female sex | 0.5 (0.1-1.9) | 0.3336 |
| C-section | 1 (0.3-4.1) | 0.9641 |
| Maternal fever during pregnancy | 1.1 (0.2-4.9) | 0.9422 |
| Maternal severe/critical COVID-19 | 1.5 (0.2-13.2) | 0.7371 |
| Maternal SARS-CoV-2 infection during the 2nd or 3rd trimester | 0.9 (0.1-6) | 0.8813 |
| Maternal mental health conditions | 1.3 (0.3-5.7) | 0.7519 |
| Other maternal comorbidities | 1.1 (0.3-4) | 0.9423 |
| Maternal age > 40 years | 6.2 (1.1-34.8) | **0.0374** |
| Infant respiratory distress syndrome (RDS) | 0.7 (0.1-4.9) | 0.6882 |

C. Both Rio de Janeiro and Los Angeles combined, Bayley Delayed < - 2SD or ASQ-3 < Cut-off

| **Variable** | **Odds Ratio (95% CI)** | **p-value** |
| --- | --- | --- |
| Low birth weight (LBW) | 0.6 (0.1-3.1) | 0.5771 |
| *SGA (Small for gestational age)* | *4.8 (0.8-27.2)* | *0.0782* |
| *Preterm delivery (<37 weeks)* | *2.5 (0.9-7.3)* | *0.0853* |
| Female sex | 1 (0.4-2.6) | 0.9781 |
| C-section | 0.7 (0.3-2.1) | 0.5685 |
| Maternal fever during pregnancy | 0.5 (0.2-1.6) | 0.2384 |
| Maternal severe/critical COVID-19 | 1.3 (0.3-4.7) | 0.716 |
| Maternal SARS-CoV-2 infection during the 2nd or 3rd trimester | 1.6 (0.4-6.7) | 0.5248 |
| Maternal mental health conditions | 1 (0.3-3.9) | 0.9989 |
| Other maternal comorbidities | 1.1 (0.4-3.3) | 0.826 |
| *Maternal age > 40 years* | *3.5 (0.8-14.8)* | *0.0827* |
| Infant respiratory distress syndrome (RDS) | 0.8 (0.2-3.9) | 0.7462 |

D. Rio de Janeiro, RJ, Brazil, Bayley Delayed or At-risk, scores < - 1SD

| **Variable** | **Odds Ratio (95% CI)** | **p-value** |
| --- | --- | --- |
| Low birth weight (LBW) | 0.9 (0.1-5.8) | 0.91 |
| Small for gestational age (SGA) | 1.5 (0.1-16.3) | 0.7453 |
| Preterm delivery (<37 weeks) | 1 (0.3-2.7) | 0.9553 |
| Female sex | 1.7 (0.6-5) | 0.3054 |
| C-section | 0.8 (0.3-2.5) | 0.7345 |
| Maternal fever during pregnancy | 0.5 (0.2-1.6) | 0.256 |
| Maternal severe/critical COVID-19 | 2.4 (0.6-9.1) | 0.2056 |
| Maternal SARS-CoV-2 infection during the 2nd or 3rd trimester | 0.5 (0.1-1.8) | 0.2676 |
| Maternal mental health conditions | 0.5 (0-17.2) | 0.7034 |
| Other maternal comorbidities | 1.3 (0.4-4.4) | 0.6789 |
| Maternal age > 40 years | 1.5 (0.1-27.7) | 0.7901 |
| Infant respiratory distress syndrome (RDS) | 1.8 (0.2-18.4) | 0.6153 |

E. Los Angeles, CA, USA, Bayley Delayed or At-risk, scores < - 1 SD or ASQ Below cut-off

| **Variable** | **Odds Ratio (95% CI)** | **p-value** |
| --- | --- | --- |
| Low birth weight (LBW) | 0.6 (0.1-7.7) | 0.7239 |
| Small for gestational age (SGA) | 2.6 (0.2-28.8) | 0.4355 |
| Preterm delivery (<37 weeks) | 1.9 (0.2-18.7) | 0.5776 |
| Female sex | 0.6 (0.2-1.8) | 0.3203 |
| C-section | 1.2 (0.3-3.9) | 0.8222 |
| Maternal fever during pregnancy | 1.1 (0.3-4.2) | 0.9122 |
| Maternal severe/critical COVID-19 | 1.9 (0.3-12.3) | 0.498 |
| Maternal SARS-CoV-2 infection during the 2nd or 3rd trimester | 1 (0.2-6.4) | 0.9855 |
| Maternal mental health conditions | 0.9 (0.2-3.6) | 0.8503 |
| Other maternal comorbidities | 0.8 (0.2-2.5) | 0.6421 |
| Maternal age > 40 years | 4.2 (0.8-21.4) | *0.08* |
| Infant respiratory distress syndrome (RDS) | 1.1 (0.2-6.4) | 0.8753 |

F. Both Rio de Janeiro and Los Angeles combined, Bayley Delayed or At-risk or ASQ Below Cut-off

| **Variable** | **Odds Ratio (95% CI)** | **p-value** |
| --- | --- | --- |
| Low birth weight (LBW) | 0.7 (0.2-2.1) | 0.4812 |
| Small for gestational age (SGA) | 2.4 (0.5-10.6) | 0.2455 |
| *Preterm delivery (<37 weeks)* | *2.1 (1-4.6)* | *0.0603* |
| Female sex | 0.9 (0.4-1.8) | 0.7244 |
| C-section | 1 (0.5-2.1) | 0.9808 |
| Maternal fever during pregnancy | 0.8 (0.4-1.8) | 0.6659 |
| **Maternal severe/critical COVID-19** | **2.6 (1.1-6.4)** | **0.0327** |
| Maternal SARS-CoV-2 infection during the 2nd or 3rd trimester | 0.7 (0.3-1.9) | 0.4928 |
| Maternal mental health conditions | 0.4 (0.1-1.2) | 0.1129 |
| Other maternal comorbidities | 0.7 (0.3-1.6) | 0.4503 |
| Maternal age > 40 years | 2.4 (0.7-8.4) | 0.1765 |
| Infant respiratory distress syndrome (RDS) | 1.1 (0.3-3.6) | 0.9039 |

1. p-values < 0.05 are bolded, and those < 0.1 are italicized

2. SARS-CoV-2 vaccination was not included in the logistic regression because the timing of immunization of pregnant women in Rio de Janeiro started later than in Los Angeles, making it difficult to compare this variable between the sites.
